# Oxidative Stress and Cardiovascular Dysfunction: From Basic Science to Applied Investigations

**DOI:** 10.1155/2020/6985284

**Published:** 2020-07-19

**Authors:** Vladimir Jakovljevic, Dragan Djuric, Olga Pechanova, Sergey Bolevich, Suresh Tyagi

**Affiliations:** ^1^Department of Physiology, Faculty of Medical Sciences, University of Kragujevac, Kragujevac, Serbia; ^2^Department of Human Pathology, 1st Moscow State Medical University IM Sechenov, Moscow, Russia; ^3^Faculty of Medicine, Institute of Medical Physiology “Richard Burian”, University of Belgrade, Belgrade, Serbia; ^4^Institute of Normal and Pathological Physiology, Centre of Experimental Medicine, Slovak Academy of Sciences, Bratislava, Slovakia; ^5^Department of Physiology, University of Louisville, Louisville, Kentucky, USA

The editors of the present special issue are pleased to introduce to the readers the overview of basic and applied research articles gathered around the connection between oxidative stress and cardiovascular dysfunction. The topic of this issue belongs to the new current and inexplicable category. Its fascination lies in the fact that despite the incredible number of researches that have provided answers to many questions in recent decades, they have simultaneously enabled the opening of new questions and dimensions of this topic. Explanations for this is reflected in the molecular mechanisms underlying the cardiovascular damage induced by oxidative stress. Exactly through the growing investigation of this field, we have noticed that these mechanisms are extremely complex and extend in all directions. So far, it has been confirmed that oxidative stress triggers endothelial dysfunction and therefore, it poses a pivotal role in all stages of atherosclerotic plaque formation [1–3]. Accumulated data provide a strong evidence that prior to definitive disorders in the vascular wall, there is a high correlation between the quantity of oxidative load and proportion of vascular dysfunction [4, 5].

The articles of this special issue brought novel information from both sides of this matter, basic and clinical. The starting point for all oxidative stress-induced cardiovascular pathological processes is endothelial dysfunction. The intercellular junctions are key factors for a maintenance of endothelium integrity. Among other effects in the endothelium, free radicals cause disruption of these junctions and thus diminish endothelial barrier integrity and function. Having in mind that molecular mechanisms of these changes are still unclear, H. Wang and coauthors conducted their research entitled “Gamma Radiation-Induced Disruption of Cellular Junctions in HUVECs Is Mediated through Affecting MAPK/NF-*κ*B Inflammatory Pathways.” By using human umbilical vein endothelial cells (HUVECs), they showed that gamma radiation-induced oxidative and nitrosative stress leads to junction's damages through activation of the MAPK/NF-*κ*B signaling pathway. However, these effects can be abolished at least in part by inhibition of the NF-*κ*B signaling pathway.

Even nitrovasodilators may cause endothelial dysfunction by induction of oxidative stress as investigated by S. Steven and coworkers in their study “The Endothelin Receptor Antagonist Macitentan Improves Isosorbide-5-Mononitrate (ISMN) and Isosorbide Dinitrate (ISDN) Induced Endothelial Dysfunction, Oxidative Stress and Vascular Inflammation”. It was found that these adverse effects of nitrovasodilators are mediated via NOX-2/ET-receptor signaling axis leading to increased vascular oxidative stress and inflammation. They have shown for the first time that dual ET receptor antagonist macitentan improves and can abolish these negative impacts of the organic nitrates and thus made an excellent basis for further clinical investigations.

In addition to the well-known inductors of endothelial oxidative stress, in recent years, it was pointed out that monoamine oxidases (MAO) can also promote reactive oxygen species (ROS) generation in both vessels and the heart. This was mentioned in a minireview made by A. Sturza et al. entitled “Monoamine Oxidase-Related Vascular Oxidative Stress in Diseases Associated with Inflammatory Burden.” This paper offered valuable data referring to the beneficial impact of MAO inhibitors in conditions associated with oxidative stress and inflammation, such as hypertension, metabolic disorders, and chronic kidney disease.

Nevertheless, if we move a little beyond the boundaries of the endothelium, we can also find factors and tissues that affect vascular morphology and function. During the last years, high attention has been paid on perivascular adipose tissue (PVAT). Namely, it has been documented that PVAT generate several molecules whose common characteristic is hyperpolarization of cell membrane and thus inhibition of vasoconstriction. A. Zemančíková and J. Török in their study “Influence of Age on Anticontractile Effect of Perivascular Adipose Tissue in Normotensive and Hypertensive Rats” have found that PVAT-induced inhibition of vasoconstriction does not depend on hypertension conditions. Moreover, it seems that the anticontractile effect of PVAT foregoes rise in blood pressure and might influence the development of hypertension.

On the other hand, oxidative injuries to the coronary endothelium are specific and clinically highly significant primarily considering the tissue they are supplying. Oxidative stress stimulates inflammasome activation which consequently induces adverse inflammatory responses after myocardial ischemia/reperfusion (I/R). In addition, a key target for controlling inflammasome is nucleotide-binding oligomerization domain-like receptor with a pyrin domain (NLRP3). A potentially novel agent against oxidative and inflammatory damages of the endothelium is stabilized ester form of the oxidant pyruvate called ethyl pyruvate (EP). J. H. Jun and colleagues investigated this field in a research entitled “Protective Effect of Ethyl Pyruvate against Myocardial Ischemia Reperfusion Injury through Regulations of ROS Related NLRP3 Inflammasome Activation.” They have shown that the protective effect of EP on rat myocardial infarction is achieved by blockade of NLRP3 inflammasome activation. Previous studies on myocardial I/R injury proved the role of transient receptor potential vanilloid 4 (TRPV4) cation channel in heart ROS generation. The study of Q. Wu et al. entitled “Blockade of Transient Receptor Potential Vanilloid 4 Enhances Antioxidation after Myocardial Ischemia/Reperfusion” assessed the antioxidative potential of TRPV4 inhibitors in *in vitro* and *ex vivo* models of heart injuries. In summary, the authors concluded that TRPV4 inhibitors activate the Akt/Nrf2/ARE pathway and improve antioxidative enzyme activity thus exerting cardioprotective effects. Furthermore, another article of this special issue entitled “Excessive Neutrophil Extracellular Traps Formation Aggravates Acute Myocardial Infarction Injury in Apolipoprotein E Deficiency Mice via ROS-Dependent Pathway” accentuates a new role of ROS in myocardial I/R. In this extensive study, Z. Zhou and colleagues showed that lack of apolipoprotein E stimulates neutrophil extracellular traps (NETs) via activation of ROS generation and MAPK-MSK1 signaling pathways. Moreover, inhibition of NADPH oxidase can limit NET formation and be a novel approach in cardioprotection.

Besides pharmacological treatment of oxidative-induced myocardial I/R, there are excellent papers suggesting involvement of plants or plant-derived active principles. The first paper “Effects of Curcumin Nanoparticles in Isoproterenol-Induced Myocardial Infarction” by P.-H. Boarescu and colleagues investigated the effects of curcumin nanoparticles on isoproterenol- (ISO-) induced myocardial infarction in rats. The authors have particularly emphasized the importance of nanotechnology in improving the bioactivity of curcumin. Indeed, their results indicate that curcumin nanoparticles ameliorate antioxidant mobility and therefore diminish myocardial injury during I/R. Similarly, J. Bradic and coauthors in study “Protective Effects of Galium verum L. Extract against Cardiac Ischemia/Reperfusion Injury in Spontaneously Hypertensive Rats” examine the effects of methanol extract of G. verum on myocardial I/R injury in spontaneously hypertensive rats. They highlighted that G. verum improved systemic redox state and modulate cardiac generation of prooxidants thus alleviating oxidative stress-induced heart damage.

It has been documented that modified redox equilibrium is an important mechanism in the pathogenesis of diabetes mellitus and its cardiovascular complications. Considering that folic acid is a well-established antioxidative supplement, S. Mutavdzin et al. in the investigation “The Effects of Folic Acid Administration on Cardiac Oxidative Stress and Cardiovascular Biomarkers in Diabetic Rats” analysed the impact of this vitamin on the redox status of diabetic rats. Their results have shown that folic acid is highly capable to enhance antioxidative enzyme activity and therefore increase antioxidative protection in diabetic conditions.

All of these basic investigations seek to find novel mechanisms involved in oxidative stress-related disorders of the vessels and heart and represent valuable basis for applied investigations. However, clinical studies in the present special issue also bring noteworthy findings. D. La Russa et al. in the research entilted “Oxidative Balance and Inflammation in Hemodialysis Patients: Biomarkers of Cardiovascular Risk?” estimated redox status as the main link in complex interaction between cardiovascular disease (CVD) and chronic kidney disease (CKD). According to results of this study, it can be noticed that hemodialysis patients with cardiovascular pathologies are connected with higher values of both oxidative stress and antioxidant defense compared to those without cardiovascular events. Hence, the maintenance of an adequate redox balance may be a new therapeutic access for preventing cardiovascular complications during CKD progression.

Chronic venous insufficiency (CVI) is another cardiovascular disorder associated with oxidative stress. In recent decades, much effort has been done to find an effective antioxidant capable to neutralize oxidative damages within the vein vasculature. Among novel phototherapy agents, a natural flavone diosmin has appeared lately with a promising antioxidant potential. In a study “Influence of Diosmin Treatment on the Level of Oxidative Stress Markers in Patients with Chronic Venous Insufficiency” made by M. Feldo and coworkers, exactly diosmin has been investigated in patents with CVI. Through measuring of isoprostanes, the authors have shown the vigorous ability of diosmin in alleviating oxidative stress in these patients.

At the end, it is certain that all mentioned above supports our initial remark regarding the depth and breadth of the relationship between oxidative stress and cardiovascular dysfunction. The goal of this special issue was therefore to provide contribution for better understanding of this topic. The editors hope that compendium of the current research in this field will be of interest to the readers and help in the creation of further investigations.
